# Melon Root

**Published:** 1887-12

**Authors:** 


**Melon Root.**—Melon root is the latest rival of ipecac. The active principle is called melon emetine, by Torosicviez, its discoverer.—*Medical Digest.*

